# Severe Rotational Drift of an Impacted Mandibular Third Molar: A Case Report

**DOI:** 10.5681/joddd.2010.025

**Published:** 2010-09-16

**Authors:** Ehsan Abouei Mehrizi, Mostafa Esmaeili, Gholamreza Eslami Amirabadi, Mohammadamin Narimani

**Affiliations:** ^1^ Post-graduate Student, Department of Orthodontics, Faculty of Dentistry, Shahed University, Tehran, Iran; ^2^ Post-graduate Student, Department of Oral Medicine, Faculty of Dentistry, Shahed University, Tehran, Iran; ^3^ Assistant Professor, Department of Orthodontics, Faculty of Dentistry, Shahed University, Tehran, Iran

**Keywords:** Impacted, mandibular, rotational drift, third molar

## Abstract

Third molar eruption is an unpredictable event. Occasionally, mandibular third molars undergo angular changes in young adults. This rotational drift is of unknown etiology, is not predictable and may facilitate the eruption or may make the im-paction worse. A rare case is presented with severe rotational drift of a mesioangularly impacted mandibular third molar.

## Introduction

Third molar eruption is an unpredictable event. The average age for eruption of third molars is 20 years, although eruption may continue in some patients until the age of 25.^1^ During growth and development ramus remodels posteriorly and resorption of its anterior surface creates space for the sequential eruption of primary and permanent molars; however, this growth ceases before enough space is created for eruption of third molars, which become impacted in the ramus.^2^ Third molar has the greatest incidence of impaction in the dentition. Most observed impactions of mandibular third molars are mesioangular, which fully erupt less than distoangularly impacted third molars.^1^

There is controversy between the advocates of early removal against follow-up of asymptomatic impacted third molars. Observation rather than prophylactic removal is based on the complete eruption of some apparently impacted third molars in young adults; in the mandible, with the exception of horizontally impacted third molars, a substantial proportion of impaction types do erupt fully. The second reason is the low incidence of pathologic conditions associated with retained third molars.^1 , 3 , 4^ On the other hand, proponents of early removal emphasize the preventive potential of early removal and avoiding the complications associated with surgery on a fully-developed third molar.^4^ Deliberate preservation of an impacted third molar, however, carries a risk of pathologic sequelae, including cyst and tumor formation, root resorption and periodontal problems of the second molar.^5^

There is no consensus on the methods to predict the behavior of mandibular third molars. Olive and Basford^6^ used the ratio between inter-second molar width and inter-ramal width on posteroanterior cephalograms. Richardson found that the original angulation of the occlusal surface of the third molar to madibular plane is significantly lower in early-erupting third molars but according to a review article this is not a promising method.^5^

## Case Report

**Figure 2:**
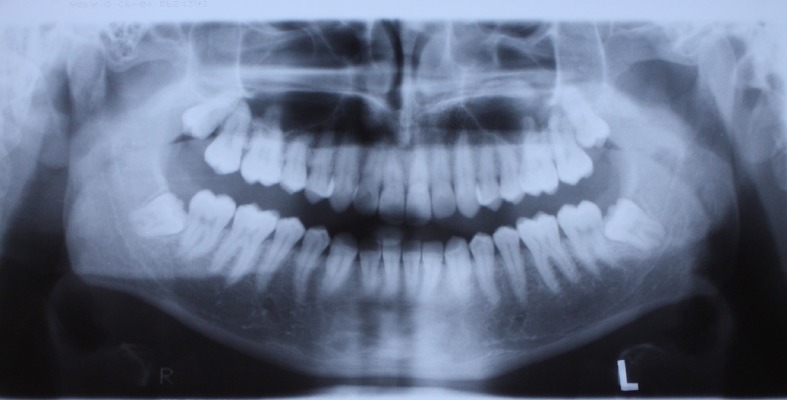
Panoramic radiograph taken 6 years ago.

A 29-year-old male who sought orthodontic treatment was referred to the Orthodontic Clinic of the Dental School of Shahed University, Tehran, Iran in 2010. He had no medical history of systemic disease. There were no caries and periodontal problems. His face was symmetric with Class I dental relationship and mild crowding in both arches. In radiographic assessment a deeply impacted horizontal mandibular third molar was found on the right side, which was directed toward the apex of the second molar (Figure 1). The patient did not have any signs and symptoms. The patient had a panoramic radiograph of 6 years ago, presenting four impacted third molars, with mesioangular lower third molars (Figure 2). The left upper and lower third molars had been surgically removed then because of a periodontal problem in the mandibular second molar area and acceptable healing was evident. The right lower third molar was retained because the patient had no symptoms; it had not erupted after 6 years and during the period had experienced a severe rotational drift of approximately 90 degrees, as compared with its recent panoramic and lateral cephalometric views. The degree of rotation was far beyond the range of image variations between different radiographic machines.


Figure 1. Panoramic (a) and lateral cephalometric (b) radiographs.

**a:**
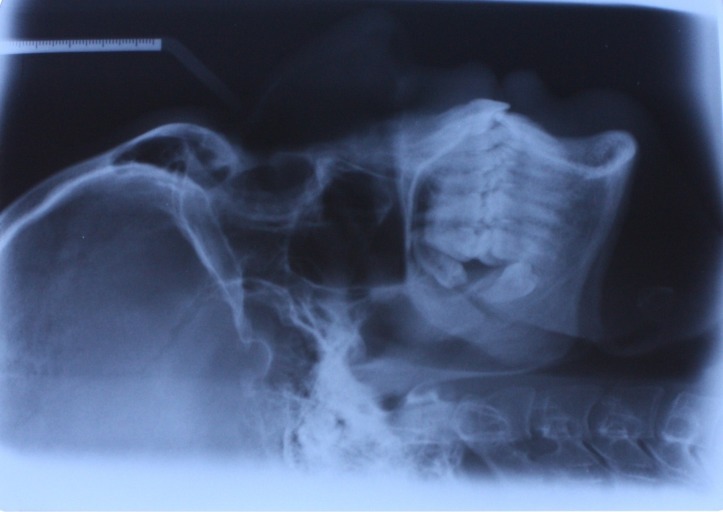

**b:**
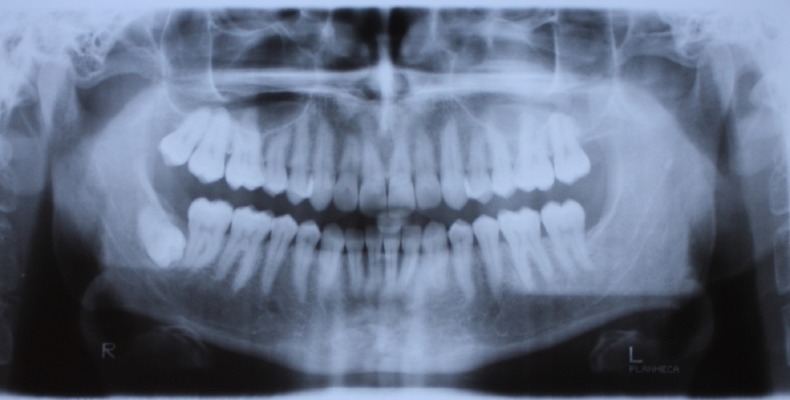

There was no sign of pathologic conditions in his clinical history and radiographs. In addition, at surgical removal, tissues were sent for histopathologic examination and no pathologic lesions were reported in association with the third molar.

## Discussion

Some authors have reported cases of lower third molar rotation throughout the second and third decades of life.^4 , 8^ It is not possible to draw conclusions from current data about the incidence and etiology of this rotation.

Sandhu & Kaur^3^ in a 4-year follow-up of 43 subjects with a mean age of 19 observed that 15% of mandibular third molars changed their sagittal inclination.According to Shiller^9^ a significant number of mesioangular impactions of mandibular third molars in young adults attain an upright position in a year. A significant relationship exists between the initial age of the patient and initial inclination on one hand and the amount of rotation on the other.

During a median of 2.2-year follow-up by sequential panoramic radiographs of 237 patients, 26% of impacted mandibular third molars changed angulation or position; 16% of mandibular mesial/horizontal impacted third molars became vertical/distal (11% erupted to the occlusal plane and 5% did not).^10^

It is claimed that severe aggressive periodontal disease of adjacent molars might result in the third molar rotational drift.^8^ Nance et al^10^ reported that erupting third molars are more likely to have probing depths more than 4 mm; in her opinion pericoronal pathologic lesions (e.g. dentigerous cysts and hyperplastic dental follicles) could contribute to the etiology. Pathologic conditions such as cysts and tumors are believed to displace mandibular third molars but in the absence of pathologic conditions the etiology is obscure.^8^ Root development might cause eruptive potential, altering tooth position.^8^ Studies have not evaluated this factor in conjunction with rotational drift phenomenon. In the presented case root formation was significantly advanced on the previous radiograph; therefore, it is difficult to explain this phenomenon after root development.

Hattab^4^ evaluated changes in angular position and eruption status of mesioangularly impacted mandibular third molars by panoramic radiographs: 42% of them fully erupted to occlusion and a significant number changed their angulation to reach full eruption by age 24. In his opinion, changes in angular position and eruption of impacted mandibular third molars are unpredictable.

Hughes et al^8^ presented four cases of extreme rotational drift of impacted mandibular third molars. All the teeth were initially mesioangular and then rotated to a horizontal or distoangular impaction. Root formation was significantly advanced and no pathologic lesions were evident.

One possible explanation might be the facial growth that continues during adult life and the associated remodeling of ramus.^2^

In the presented case, 6 years of unnecessary delay in treatment had led to severe rotational drift of a mesioangularly impacted lower third molar by an unknown mechanism, which had complicated the surgical procedure.

## Conclusion

Knowledge of the fate of mandibular third molars after early adulthood is required to make a correct decision about the treatment of asymptomatic impactions. The traditional assumption that mandibular third molars do not change their position after hard tissue impaction in the third decade of life is rejected.

Possible undesirable rotation in asymptomatic lower third molars is a reason for early treatment. In some cases unnecessary delay in the treatment may worsen the situation and make surgery more difficult and complicated, particularly when the patient gets older. If surgical removal of impacted lower third molars is not intended, they should be regularly followed in the second and third decades of life.
